# Towards AI-powered personalization in MOOC learning

**DOI:** 10.1038/s41539-017-0016-3

**Published:** 2017-12-14

**Authors:** Han Yu, Chunyan Miao, Cyril Leung, Timothy John White

**Affiliations:** 1 Joint NTU-UBC Research Centre of Excellence in Active Living for the Elderly (LILY), Nanyang Technological University, Singapore, 639798 Singapore; 2 School of Computer Science and Engineering, Nanyang Technological University, Singapore, 639798 Singapore; 3 Department of Electrical and Computer Engineering, The University of British Columbia, Vancouver, BC V6T 1Z4 Canada; 4 School of Materials Science and Engineering, Nanyang Technological University, Singapore, 639798 Singapore

## Abstract

Massive Open Online Courses (MOOCs) represent a form of large-scale learning that is changing the landscape of higher education. In this paper, we offer a perspective on how advances in artificial intelligence (AI) may enhance learning and research on MOOCs. We focus on emerging AI techniques including how knowledge representation tools can enable students to adjust the sequence of learning to fit their own needs; how optimization techniques can efficiently match community teaching assistants to MOOC mediation tasks to offer personal attention to learners; and how virtual learning companions with human traits such as curiosity and emotions can enhance learning experience on a large scale. These new capabilities will also bring opportunities for educational researchers to analyse students’ learning skills and uncover points along learning paths where students with different backgrounds may require different help. Ethical considerations related to the application of AI in MOOC education research are also discussed.

## Introduction

Massive Open Online Courses (MOOCs), which make university courses available at nominal or no cost to thousands of students, are a disruptive technology challenging traditional educational models. With the ability to reach a large number of learners around the world, MOOCs have made a positive impact on open education. However, scaling up class sizes while maintaining quality is not easy, and many problems remain.^1^ Communication and information diffusion through MOOCs are limited by how learners connect both in the courses and through other social networks.^2^ Learners often complain that current MOOCs fail to provide enough hands-on experience to help them translate concepts and ideas into practical skills.^3^ The dropout rates for many MOOCs remain elevated.^4^

Today, it is well-recognized that effective learning through MOOCs requires pedagogies different from those used in face-to-face learning.^5^ In order to cater to students with different backgrounds, personalization is especially important for effective learning.^6^ In MOOCs, students generate rich learning behaviour data through interactions with online learning contents.^7^ For years, people have been trying to find ways to apply artificial intelligence (AI) techniques to unlock value from these data (e.g. identifying who are likely to drop out of a course early on so that more attention be given to those who are likely to remain^8^). Nevertheless, the application of AI in MOOCs is not always straightforward. The main reason is likely to be that current MOOC teaching is still more focused on standardization, not personalization. From learning contents to tests, MOOCs today largely resemble classroom teaching where students fit within pre-determined parameters that leave little room for individuality, creativity or critical thinking.

As MOOC platforms (e.g. Coursera, edX) have not yet made learning behaviour data across multiple courses accessible to AI researchers,^9^ existing AI research in this area mostly relies on course-specific datasets released in an ad-hoc manner (e.g. the KDD Cup 2015 competition dataset). In order to support open science in education through MOOCs, MOOC platforms need to collaborate with educators and AI researchers. In this paper, we offer a perspective on how advances in AI may support the personalization of learning at a large scale and enhance research in MOOCs. We focus on emerging AI techniques, including how knowledge representation tools can enable students to adjust the sequence of learning to fit their own needs; how optimization techniques can efficiently match community teaching assistants to MOOC mediation tasks to offer personal attention to learners; and how virtual learning companions (VLCs) with human traits such as curiosity and emotions can enhance learning experience on a large scale. These new capabilities will bring new opportunities for educational researchers to analyse students’ learning skills and uncover points along learning paths where students with different backgrounds may require different help. Ethical considerations related to the application of AI in MOOC education research are also discussed.

## Personalizing learning activities

As a form of e-learning, the use of online resources provides MOOCs with a natural means to deliver personalized learning opportunities to students. For example, the use of online mini-libraries together with course materials allows interested students to further explore related topics.^10^ Current MOOCs have fixed course structures, in which, for example, the sequence of video lectures and related learning activities are pre-defined by course designers. For courses on subjects with clearly defined dependence among the topics (e.g. normally learning to perform addition comes before learning to perform subtraction in mathematics), letting all students follow the same learning path is advantageous. However, for courses on some advanced topics (e.g. human–computer interaction), the dependence among topics is not always clearly defined. As MOOCs are often university level courses, many belong to the latter category. For these types of courses, it may be advantageous to allow students more freedom to access the learning contents without strictly following the pre-defined sequence based on their personal situations (e.g. background knowledge, time constraints). There is also evidence suggesting that educators can benefit from learners’ inputs on the sequence of learning contents in e-learning environments.^11^

In order to provide future MOOC learners with the flexibility to personalize their learning paths, the first step is to refine learning contents into finely grained and well labelled knowledge units. The technique of backward design,^12^ in which course instructors decide objectives, determine evaluation criteria and then choose learning activities, can be used to develop courses with finely grained coherent learning contents. An emerging trend in online learning—*micro-MOOCs*—offers a form of MOOCs which allows course instructors to implement backward design more conveniently. They employ a new form of micro-lectures which typically consist of short videos on specific topics, typically no longer than 5 min each, along with concise textual explanations.^5^ Using micro-MOOCs as the main form of learning content delivery, new MOOC service providers such as *Curious*, *Everpath* and *Pathwright* are attracting students who have short and fragmented time for MOOC learning (e.g. working professionals).

**Fig. 1:**
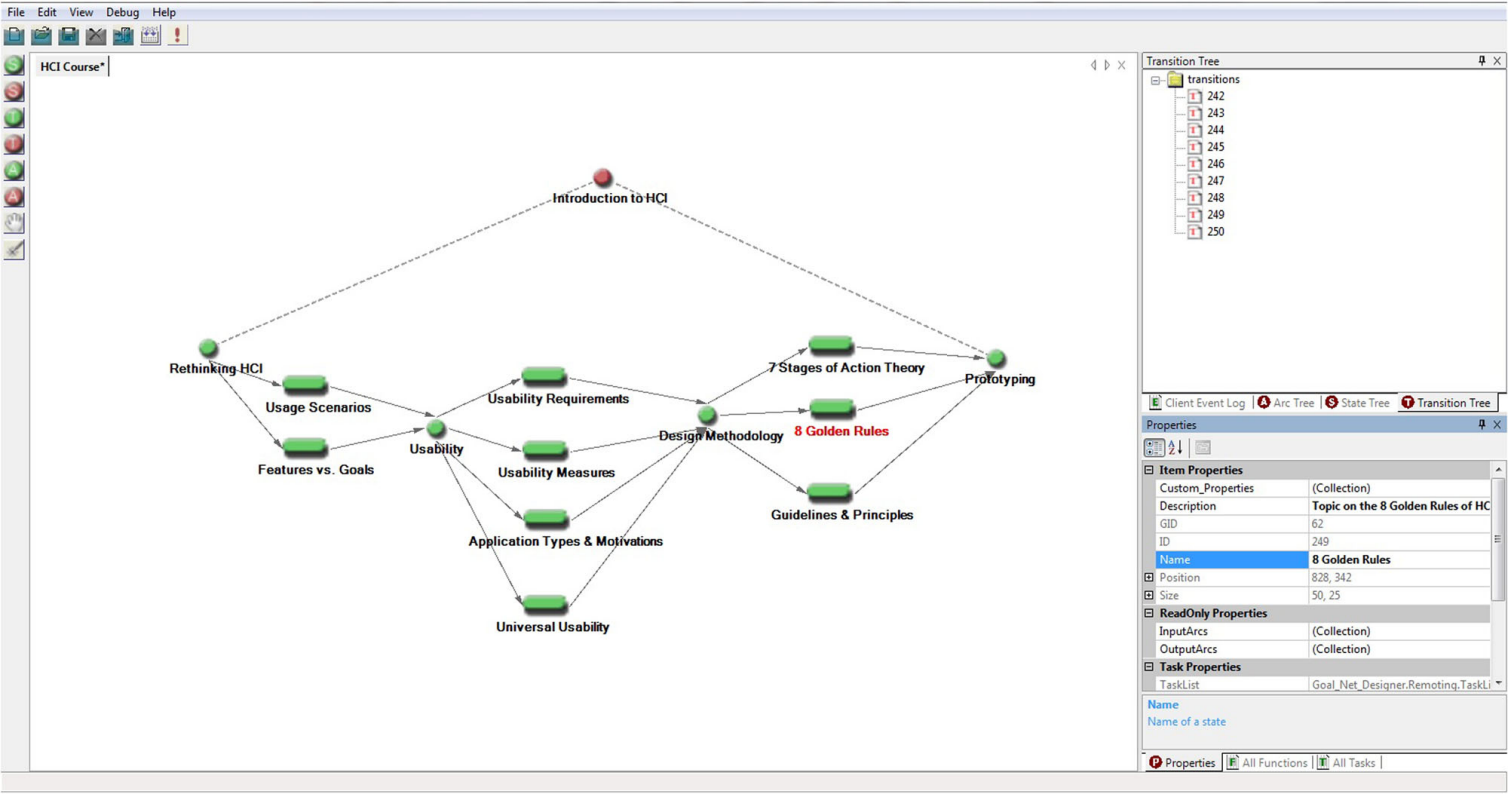
Modelling a learning path for the Introduction to Human-Computer Interaction (HCI) course using Goal Net. The software shown in this figure was created by the authors who own the copyright

With micro-MOOCs serving as potential building blocks of learning paths, the next step requires easy-to-use software tools to construct and modify learning paths. A candidate for such a tool is the *Goal Net* methodology and the associated *Multi-Agent Development Environment (MADE)*.^13^ It is an AI knowledge representation tool designed to model logic flows which can guide the actions of software agents in a visual way without having to write lengthy programme codes.^14^ Based on the goal setting theory,^15^ Goal Net allows users to express a complex process (e.g. organizing a set of learning tasks) as a network of goals and activities. It also enables transitions among goals and the achievement of goals to be tracked. Figure 1 shows an example of a learning path consisting of learning activities (rounded rectangles) and learning goals (circles) involved in the Introduction to Human-Computer Interaction (HCI) course built using Goal Net. In this course, some topics such as 'Application Types & Motivation', 'Universal Usability', '7 Stages of Action Theory', '8 Golden Rules' and 'Guidelines & Principles' do not have strong dependencies on each other and can be taken in different order. The learning activities can take the form of micro-MOOCs, quizzes, forum discussions and other learning instructional formats.

Using a combination of micro-MOOCs and learning path construction tools (possibly derived from Goal Net or other similar tools such as the Belief-Desire-Intention (BDI) model^16^), instructors can specify a general syllabus consisting of finely grained learning contents and activities with improved efficiency and flexibility of updating. In courses in which the dependency among topics is not strong, the learning path construction tool could be open for MOOC learners to use in order to personalize individuals’ learning paths. This will not only help learners keep track of their personalized learning paths during the course but also serve as a knowledge representation format to record the various personalized learning paths in a machine understandable way. When we analyse the correlations between the personalized learning paths, students’ background information, and their learning outcomes (which have already been recorded by existing MOOC platforms) with machine learning, this envisioned mechanism may offer educational researchers feedback on how to personalize learning for various courses for students from different backgrounds. Over time, this envisioned mechanism can help MOOC educators optimize learning trajectories for different courses based on learning paths which resulted in desirable learning outcomes, possibly with the help of technologies such as behaviour phenotype analytics^17^ and transfer learning.^18^

Nevertheless, the realization of this vision still has significant challenges. One of the most important is that learning outcomes are often hard to define and hard to measure (e.g. are a high test scores indicative of good learning outcomes?). Without proper definition of a learning outcome objective function, machine learning approaches will not be able to compute optimized personal learning paths. In addition, as the available learning behaviour data are mostly from people who managed to complete the MOOCs, it is possible that the AI models trained from such data may be biased towards high performing learners. The extent to which learners may be affected by this possible bias requires more studies to be fully understood.

## Personalizing learning support

It can be argued that the current MOOC platform infrastructure is designed to efficiently help students 'learn facts' rather than 'acquire skills'. This design is suitable for courses which are heavy on knowledge (including facts, theories and formulae). For this type of courses, assessment of learning outcomes can be done objectively based on quizzes and examination scores. However, there are courses which emphasize skills (application of knowledge). For such courses in which students are to acquire skills, the current mechanism of content delivery and assessment in MOOC platforms is not very suitable. Rubrics^23^ derived from learners’ performance in learning tasks reflect students’ learning skills to some extent, but are incomplete. Interactions between course instructors and students as well as among students of the same course could provide opportunities to generate complex behaviour data which can be used to analyse students’ learning skills (e.g. twenty-first century learning skills such as collaboration with others, time management and critical thinking^19^). The analysis results can supplement quiz/exam-based assessment to support smart intervention mechanisms (e.g. real-time hints or feedbacks^20^) or to alert course instructors on specific weaknesses in some students with regard to certain learning skills.

Currently, MOOC platforms provide several tools for instructional designers to incorporate interactions with learners into courses. These tools typically include discussion boards, live chats, small group classrooms and project based learning. However, running such interactive sessions require time and effort which becomes unfeasible for MOOCs with large student populations. Techniques such as involvement of community teaching assistants (TAs),^21^ peer assessment tools,^22^ well-designed elaborated rubrics with specific content feedback^23^ and video feedbacks^24^ are available to help MOOC instructors scale up the provision of perceived personal attention to students. Data from these interactions have not yet been systematically collected and managed to support assessment of learning skills,^49^ however. In addition, as the choice of incorporating these interaction techniques into a MOOC is up to the course instructor, the use of these techniques across courses varies. Currently, data concerning learner interaction behaviours in MOOC tend to be sparse. Although AI techniques such as Generative Adversarial Networks^25^ can partially address the data sparsity issue in pattern recognition tasks by synthesizing artificial training data based on the statistical distribution of a small amount of real training data, if they were trained on sparse available learning behaviour data, there is still the risk that the resulting generated data will not capture reality well. Using data derived from interaction mechanisms currently available in MOOC platforms, machine learning-based learner assessment techniques mainly reflect how students interact with, rather than learn from, the courses.^9,26,27^ It is possible to apply emerging topic-modelling-based machine learning approaches to infer learners’ attitude, ability and latent skill levels based on forum discussion data if the forum is actively used by learners.^28^ Thus, creating more interaction opportunities for learners to exhibit various learning skills would help generate the data needed for AI to accurately assess learners’ skills.

AI could help enhance interactions with MOOC learners while generating much needed learning behaviour data to facilitate learning skills analytics in two main ways. In MOOCs, community TAs often participate on a volunteer basis. In the short term, AI techniques can be applied to scale up and make efficient utilization of the collective productivity of community TAs through stochastic optimization, in which data-driven algorithmic management can optimize work division and scheduling. Currently, they are recruited on a small scale (typically less than 10 community TAs per course) and mainly perform discussion moderation tasks.^21^ Even so, it has been reported that most community TAs (about four out of five) declined invitations to perform community TA duties for a second time.^29^ These studies found that the small scale and poor retention of community TAs limit interactions and attentions which can be provided to MOOC learners. Stochastic optimization maximizes or minimizes objective functions or constraints which involve random variables. In the domain of crowdsourcing, data-driven algorithmic management approaches based on stochastic optimization have been used to dynamically divide work among workers so as to fairly allocation workload and achieve superlinear collective productivity,^30–34^ schedule rest-breaks^35^ and adjust incentives to encourage participation based on game theory.^36,37^ If MOOC platforms could incorporate mechanisms allowing learners who have interacted with a community TA to provide ratings on the TA’s performance, these approaches could be applied in the context of MOOCs to scale up the involvement of community TAs and make more efficient use of community TAs’ collective effort.

In the long term, a potential AI-based solution towards interactive learning experience at a large scale is VLCs.^38^ Currently, Jill Watson,^39^ an AI teaching assistant which is built on top of the IBM Watson platform, is helping the Knowledge Based Artificial Intelligence MOOC offered by Georgia Institute of Technology to answer students’ questions. In order to provide believable interactions, VLCs need to be equipped with human-like characteristics. A curious companion was proposed^40,41^ to provide personalized guidance to learners in a virtual world-based learning environment. By monitoring the learner’s progress, the curious companion identifies learning contents that can potentially arouse the learner’s curiosity so as to maintain the learner’s interest. A remembrance companion was proposed to help learners deal with large volumes of knowledge.^42^ Modelled on how humans organize and recall information from memory, the remembrance companion helps learners recall previously acquired knowledge that is related to the current learning task in real time. To avoid over-reliance by a learner on the remembrance companion, it interacts with the learner through progressively more explicit hints. A teachable learning companion—*Betty’s Brain*—has been shown to help learners consolidate their knowledge.^43^ Based on education theories such as learning-by-teaching^44^ and argumentative learning,^45^ VLCs can help learners organize what they have learnt into coherent knowledge by facilitating discussion and seeding reflections. Interactions with VLCs can also generate learning behaviour data to facilitate learning skill analytics. As VLCs are naturally intelligent user interfaces, they can also deliver interventions to help an individual learner improve learning skills based on the analytics results.

As software agents, VLCs can address the problem of large-scale usage in MOOCs. Nevertheless, we believe it is not yet ready for two main reasons. Firstly, in order to effectively facilitate learning of complex topics instead of just answering questions, VLCs need to be able to hold meaningful conversations (either vocally or textually). However, teaching machines to converse like humans turns out to be much harder than originally anticipated. In a typical conversation, one party begins by sending the other party a signal which is acknowledged. Both parties will continue to work together to construct meaning based on a shared understanding of the topic, and constantly play back what the other party is saying against their original intentions to check if the conversation is still on track. AI conversational interaction is still an open research challenge today.

Secondly, ethical issues for incorporating VLCs into the learning process must be carefully deliberated if educational researchers wish to carry out empirical studies on their interactions with learners. As VLC research falls into the broad category of computational behavioural science, it must follow the *Belmont Report* principles in that VLCs should not violate learners’ personal autonomy, benefits brought by the VLCs should outweigh risks, and the benefits and risks should be distributed fairly among learners.^46^ As VLCs may be designed to help learners adopt more effective learning habits through learning skill analytics and smart interventions, such applications might be perceived as violating learners’ personal autonomy. Thus, mechanisms to obtain informed consent from learners will be needed in MOOC platforms which make use of VLCs. The emerging moral decision-making framework^47^ can be used to implement VLCs to reduce the risk of inappropriate use of social engineering techniques to persuade learners. Explainable AI frameworks^48^ can also be incorporated into VLCs to articulate the rationale behind their recommendations in order to build trust with learners. Nevertheless, limiting what VLCs can do in order to comply with ethical guidelines may not be the whole solution. Game theoretic modelling of adversarial behaviours^50^ should be undertaken in order to understand if learners would try to exploit these limiting considerations to game the VLCs, rendering them unable to achieve their design goals.

## Conclusion

As MOOCs are still a relatively new mode of learning, reaching for the low-hanging fruits such as advances in optimization techniques and VLCs during research and development is a reasonable strategy. The ideas discussed in this paper can enrich the MOOC designing experience. If MOOC platform operators incorporate support for personalization of learning, richer interaction opportunities and analytics of the resulting learning behaviour data into their system framework with proper ethical oversight mechanisms, MOOCs can be transformed into test-beds for advancing educational research, and ultimately, improve learning. Such a technology framework also provides a cost-effective way for findings to be quickly put to action. For these to happen, support from MOOC platform operators will be key.
